# Editorial: Innovative approaches to modulate fish gut microbiota for disease management in aquaculture

**DOI:** 10.3389/fmicb.2025.1721029

**Published:** 2025-10-31

**Authors:** Yuniel Méndez-Martínez, Ángel I. Campa-Córdova, Antonio Luna-González

**Affiliations:** ^1^Facultad de Ciencias Pecuarias y Biológicas, Universidad Técnica Estatal de Quevedo (UTEQ), Quevedo, Ecuador; ^2^Centro de Investigaciones Biológicas del Noroeste S.C., La Paz, BCS, Mexico; ^3^Departamento de Acuacultura, Instituto Politécnico Nacional (IPN), Centro Interdisciplinario de Investigación para el Desarrollo Integral Regional – Unidad Sinaloa (CIIDIR-Sinaloa), Guasave, Sinaloa, Mexico

**Keywords:** antibiotic alternatives, multi-omics, environmental stressors, pathogen genomics, sustainable aquaculture

Over the past decade, targeted modulation of the gut microbiome has moved from a theoretical promise to a pragmatic strategy for improving animal health and performance while reducing antibiotic use in aquaculture. Against this backdrop, this Research Topic brings together six contributions that, from complementary perspectives, address nutrition, phytobiotics, environmental microbial ecology, abiotic stress, and emerging pathogens with the aim of advancing management grounded in the microbial ecosystems of the host and its environment. Indeed, multiple reviews indicate that probiotics, prebiotics, and synbiotics are plausible alternatives for reducing antibiotic pressure and preventing bacterial infections in fish and crustaceans (Hoseinifar et al., 2018). Below, we synthesize these contributions, highlighting key findings, limitations, and research priorities.

It is worth emphasizing that diet remodels the intestinal ecosystem, yet the host also exhibits resilience. In this regard, in a 60 day bioassay in *Sparus aurata*, Ruiz et al. documented that an abrupt change from a commercial feed to a wild-shrimp-based diet initially increased diversity (Shannon) and altered bacterial community structure; however, by 40–60 days, communities converged and a stable core microbiome was maintained, with direct implications for substituting marine ingredients with sustainable alternatives without compromising intestinal homeostasis evidence of adaptation and resilience. Thus, fish appear to possess ecological buffering mechanisms that favor post-shift stabilization, opening the way for eco-efficient diets, provided a transition period is respected and functional effects are validated via metabolomic and immunomodulatory assessments.

An additional study in a herbivorous carp provides an integrated view of fishmeal inclusion levels, gut microbiota, and product quality. In a trial with 0, 3 and 6% inclusion, Wang et al. found significant improvements in growth and intestinal morphology at 3–6%, alongside distinct shifts in the intestinal community (NMDS separation; indicator taxa) and changes in muscle volatile compounds; notably, 6% was associated with denser fibers and a superior aroma profile, whereas 3% optimized the growth–intestine balance. Taken together, these results support balanced formulations that simultaneously weigh performance, intestinal health, and filet attributes. However, it remains to be clarified whether the modulated taxa play probiotic roles or pose a risk and how they connect to digestive efficiency and immune robustness. This line of evidence aligns with syntheses describing diet and macronutrients as primary modulators of the structure and function of the fish gut microbiome (Zhang et al., 2025).

In parallel, phytobiotics are emerging as a low-impact alternative to reinforce the three pillars: growth, immunity, and microbial resilience. In *Labeo rohita*, Yadav et al. evaluated a *Prosopis cineraria* (khejri) seed extract over 60 days followed by a challenge with *Aeromonas hydrophila*. Supplementation (optimum 5 g kg^−1^) improved growth, activated digestive and antioxidant enzymes, increased serum proteins, and reduced post-challenge mortality, evidencing an immunostimulatory effect with potential to lessen antibiotic pressure in intensive systems. As a next step, it would be valuable to map direct changes in the intestinal microbiota associated with the extract (e.g., increases in lactic-acid bacteria or butyrate producers) and to verify reproducibility at a commercial scale. The biological plausibility of these effects is supported by reviews detailing the mechanisms of action of probiotics and phytogenic additives contribute to competition for adhesion sites, competitive exclusion, bacteriocin production, and modulation of immune responses in disease prevention (Hoseinifar et al., 2018).

It has been documented that fish microbial health is not explained solely by the intestinal lumen. Evidence suggests that modulating the environmental microbiome is equally decisive. In *Litopenaeus vannamei* ponds, Huang et al. showed that adding oyster shells as substrates promotes nitrifying biofilms (dominated by *Nitrospira*), accelerates the conversion of nitrite to nitrate, and improves growth and survival an ecological, low-cost bioengineering intervention that reduces nitrogenous toxins and displaces potential opportunists in the water column. Accordingly, integrating “natural bioreactors” within the production unit can reinforce the effects of functional diets, co-creating healthy microbial landscapes in the fish and their surroundings.

It is also important to emphasize that host physiology under abiotic stress conditions affects the stability of the microbiome and the health risk. In *Apostichopus japonicus*, Cui et al. evaluated six common stressors (thermal, hyposalinity, ammonia, nitrite, crowding, and fasting) and observed performance declines and consistent dysbiosis: decreases in the *Bacteroidota:Proteobacteria* and *Firmicutes:Proteobacteria* ratios; enrichment of *Vibrionaceae* or *Shewanellaceae* depending on the stressor; and destabilization of microbial ecological networks. Notably, *Verrucomicrobia* emerged as a cross-cutting stress biomarker. Although the model is an echinoderm, the mechanistic logic of stress, dysbiosis, and susceptibility is transferable to fish and suggests the value of microbiome-based early-warning systems to trigger corrective actions (stocking density, water quality, and prebiotics/probiotics) before clinical collapse. At the innovation frontier, it has further been proposed to move toward synthetic microbiotas and the genetic engineering of commensal microbes as routes to precision therapies and pathogen control, always within robust biosafety and regulatory frameworks (Martínez-Porchas et al., 2025).

Finally, to understand the microbial adversary with genomic resolution, it is necessary to complement modulation tactics. In *Siniperca chuatsi*, Chen et al. isolated a highly virulent strain of *Nocardia seriolae* (LD_50_ = 3.89 × 104 CFU.mL^−1^), describing multiorgan granulomatous lesions and an 8.12-Mb genome harboring 403 virulence genes and multiple resistance determinants; the susceptibility profile included enrofloxacin, doxycycline, and florfenicol, among others. Beyond the specific case, these data lay the groundwork for molecular surveillance, early diagnosis, and the design of strategies that explore whether probiotic consortia or environmental manipulations can compete with or inhibit opportunistic pathogens.

Taken together, the six studies in this Research Topic converge on a clear message: a shift from reactive health management to ecosystem-based prevention, in which diet, additives, environment, stress management, and genomic surveillance are articulated to favor functional, robust microbiotas. Immediate priorities include (a) standardizing designs and metrics (including omics) for comparability across species and environments; (b) validating, at farm scale, the cost–benefit of phytobiotics, probiotics, and pro-biofilm substrates; (c) exploring fish the environment synergies (e.g., diets plus biofilms) and their effects on relative percent survival (RPS) against pathogens; and (d) translating dysbiosis markers (e.g., *Verrucomicrobia*) into operational monitoring protocols.
